# ﻿A survey of *Belisana* spiders (Araneae, Pholcidae) from eastern Sichuan, China

**DOI:** 10.3897/zookeys.1236.146511

**Published:** 2025-05-02

**Authors:** Bing Wang, Ying Wang, Shuqiang Li, Pengfeng Wu, Zhiyuan Yao

**Affiliations:** 1 College of Life Science, Shenyang Normal University, Shenyang 110034, Liaoning, China Shenyang Normal University Shenyang China; 2 Institute of Zoology, Chinese Academy of Sciences, Beijing 100101, China Chinese Academy of Sciences Beijing China

**Keywords:** Biodiversity, cellar spider, fogging, invertebrate, new record, new species, taxonomy

## Abstract

China exhibits remarkable diversity of the spider genus *Belisana* Thorell, 1898, while Sichuan Province has recorded only one species, *Belisanamaoer* Yao & Li, 2020. In this study, four species were collected in eastern Sichuan by canopy fogging. These comprise two new species, *Belisanamiyi* Wang, Li & Yao, **sp. nov.** and *B.tongjiang* Wang, Li & Yao, **sp. nov.**, as well as two known species, *B.tongi* Zhang, Li & Yao, 2024 and *B.yanhe* Chen, Zhang & Zhu, 2009, which are reported from Sichuan for the first time. A distribution map of all five species is provided.

## ﻿Introduction

**Figure 1.**
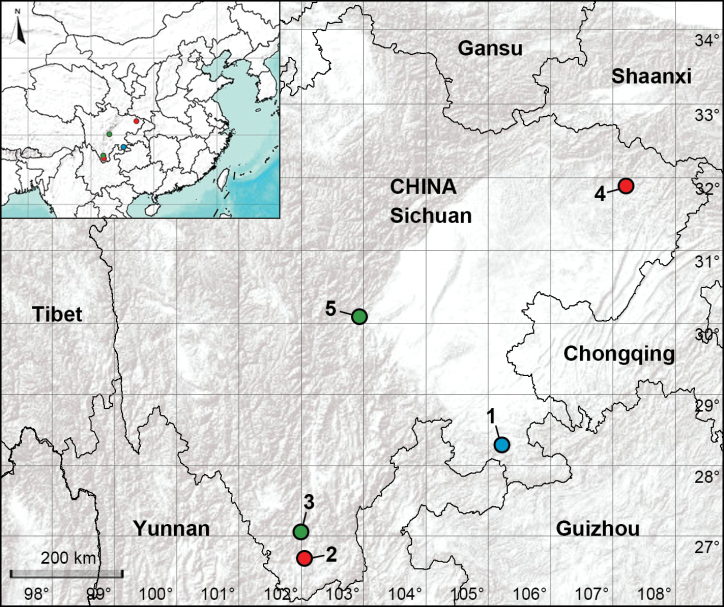
Distribution records of the *Belisana* species from eastern Sichuan, China **1***Belisanamaoer* Yao & Li, 2020 **2***B.miyi* sp. nov. **3***B.tongi* Zhang, Li & Yao, 2024 **4***B.tongjiang* sp. nov. **5***B.yanhe* Chen, Zhang & Zhu, 2009. Blue, green and red circles indicate previously recorded species, newly recorded species, and new species, respectively.

The family Pholcidae C.L. Koch, 1850 is one of the most species-rich spider families, with 2037 extant species in 97 genera (WSC 2025). *Belisana* Thorell, 1898, the second largest genus in Pholcidae, comprises 169 species (WSC 2025). They are mainly distributed in southern China, and the Indo-Malayan and Australasian regions (Huber 2005; Yao and Li 2013; Yao et al. 2018; Zhu et al. 2020). The former exhibits the highest diversity with 83 described species and represents 49% of the genus (Wang et al. 2024; WSC 2025). Recently, numerous expeditions of pholcid spiders have been conducted in China, resulting in the discovery and description of a large number of new species. These efforts have primarily focused on two genera: *Pholcus* Walckenaer, 1805 in northern and central China (e.g., Yao et al. 2021; Lu et al. 2022; Zhao et al. 2023b; Yang et al. 2024a, b; Li et al. 2025; Yao and Li 2025) and *Belisana* in southern China (e.g., Zhao et al. 2023a; Wang et al. 2024; Zhang et al. 2024a, b). Nevertheless, the distribution of these two genera in China is still conspicuously patchy. For instance, Sichuan Province, located in the southwest of China, has recorded only one species of *Belisana*: *B.maoer* Yao & Li, 2020 (Zhu et al. 2020). Sichuan is situated in the transitional zone between the Tibetan Plateau (often considered the first step of China’s geographical terrain) and the middle and lower reaches of the Yangtze River (typically classified as the third step), within the framework of China’s three major geographical steps. Furthermore, eastern Sichuan boasts a subtropical humid climate (Wang et al. 2020). Therefore, its species diversity deserves further investigation. We recently conducted a field survey targeting *Belisana* in eastern Sichuan by canopy fogging and discovered two new species. Additionally, two known species were reported from Sichuan for the first time (Fig. 1).

## ﻿Material and methods

All specimens were collected by canopy fogging. Specimens were examined and measured with a Leica M205 C stereomicroscope. Left male palps were photographed. The ventral views of epigyna were photographed before dissection. Vulvae were photographed after treating them in a 10% warm solution of potassium hydroxide (KOH) to dissolve soft tissues. Images were captured with a Canon EOS 750D wide zoom digital camera (24.2 megapixels) mounted on the stereomicroscope mentioned above and assembled using Helicon Focus v. 3.10.3 image stacking software (Khmelik et al. 2005). For each sample, ~100 individual photos were stacked together. Using Procreate v. 5.0.2 (Savage Interactive Pty Ltd), the drawings were done based on the photos, with further modifications made according to direct observations of the samples. All measurements are given in millimeters (mm). Leg measurements are shown as: total length (femur, patella, tibia, metatarsus, tarsus). Leg segments were measured on their dorsal sides. The distribution map was generated with ArcGIS v. 10.2 (ESRI Inc.). The specimens studied are preserved in 75% ethanol and deposited in the College of Life Science, Shenyang Normal University (SYNU) in Liaoning, China.

Terminology and taxonomic descriptions follow Huber (2005) and Yao et al. (2015). The following abbreviations are used: **aa** = anterior arch, **ALE** = anterior lateral eye, **b** = bulb, **ba** = bulbal apophysis, **da** = distal apophysis, **e** = embolus, **ep** = epigynal pocket, **f** = flap, **L/d** = length/diameter, **pa** = proximo-lateral apophysis, **PME** = posterior median eye, **pp** = pore plate, **pr** = procursus.

## ﻿Taxonomy

**Family Pholcidae C.L. Koch, 1850**

**Subfamily Pholcinae C.L. Koch, 1850**

### ﻿Genus Belisana

Taxon classificationAnimaliaAraneaePholcidae

Thorell, 1898

C594937F-AB28-5D7B-BC67-919663888E93

#### Type species.

*Belisanatauricornis* Thorell, 1898.

### ﻿ Belisana miyi

Taxon classificationAnimaliaAraneaePholcidae

Wang, Li & Yao sp. nov.

540FC269-A899-5947-99BA-DB14A34B5EBD

https://zoobank.org/EF9C76D6-1612-4301-87FF-3ACEAE8F7B0F

**Figure 2.**
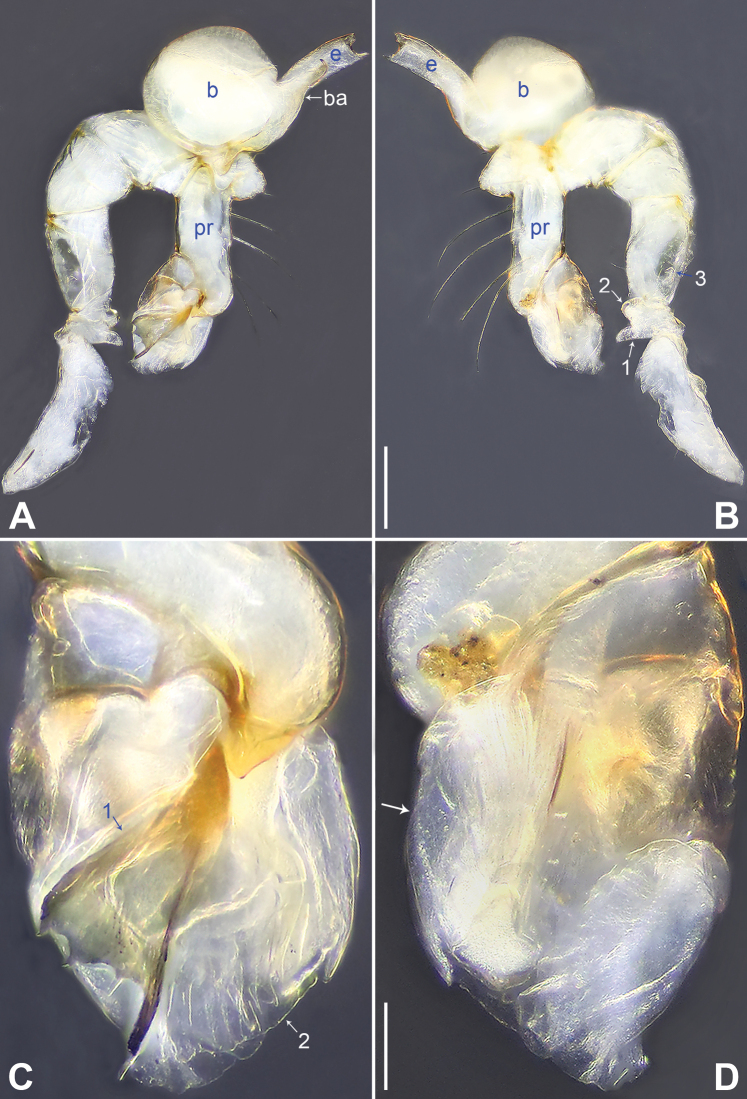
*Belisanamiyi* sp. nov., holotype male **A, B** palp (**A** prolateral view **B** retrolateral view, arrow 1 points at ventral apophysis, arrow 2 points at retrolateral apophysis, arrow 3 points at retrolateral protrusion) **C, D** distal part of procursus (**C** prolateral view, arrow 1 points at prolatero-distal sclerite, arrow 2 points at distal membranous lamella **D** retrolateral view, arrow points at retrolatero-subdistal membranous lamella). Abbreviations: b = bulb, ba = bulbal apophysis, e = embolus, pr = procursus. Scale bars: 0.20 (**A, B**); 0.05 (**C, D**).

**Figure 3.**
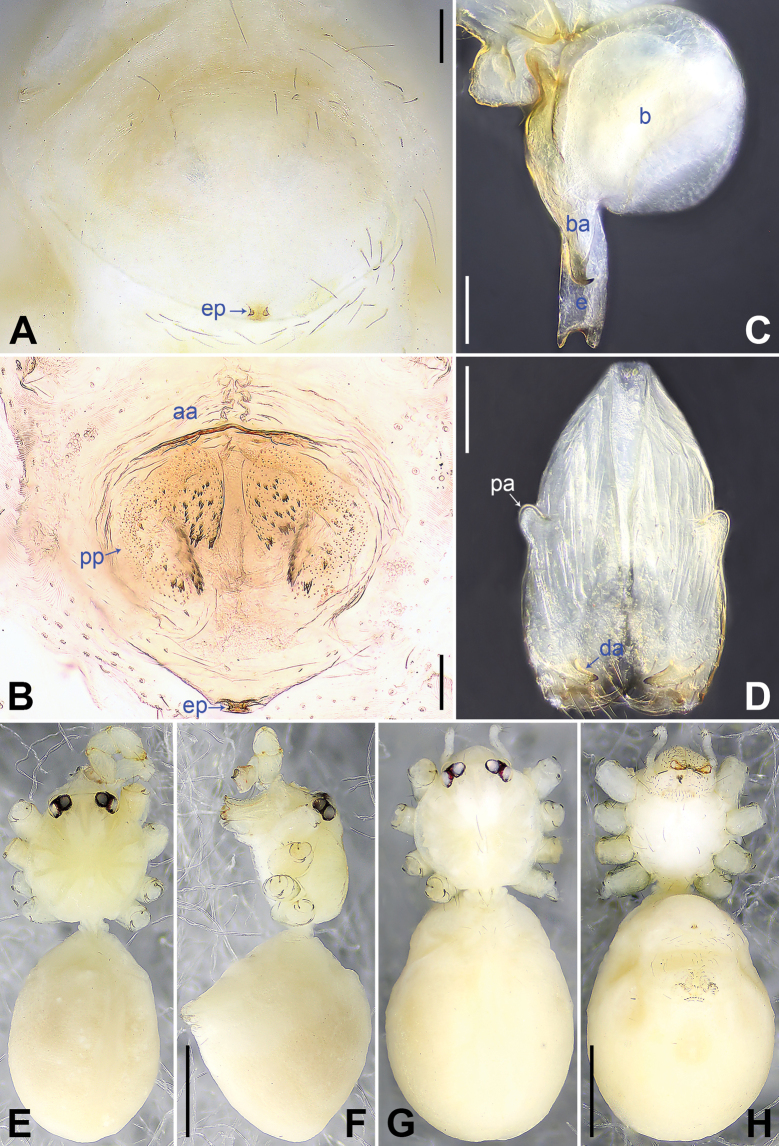
*Belisanamiyi* sp. nov., holotype male (**C–F**) and paratype female (**A, B, G, H**) **A** epigynum, ventral view **B** vulva, dorsal view **C** bulb, prolateral view **D** chelicerae, frontal view **E–H** habitus (**E, G** dorsal view **F** lateral view **H** ventral view). Abbreviations: aa = anterior arch, b = bulb, ba = bulbal apophysis, da = distal apophysis, e = embolus, ep = epigynal pocket, pa = proximo-lateral apophysis, pp = pore plate. Scale bars: 0.10 (**A–D**); 0.50 (**E–H**).

Figs 2 , 3 , 6A, B , 7A, B

#### Type material.

***Holotype*: China** • ♂; Sichuan, Panzhihua, Miyi County, Binggu Town, Maidichong Village; 26.679384°N, 102.062562°E; alt. 2235 m; 7 Jun. 2024; X. Zhang, Y. Wang & Q. Meng leg.; SYNU-Ar00453. ***Paratypes*: China** • 3 ♂; same data as for holotype; SYNU-Ar00454–56 • 1 ♀; same data as for holotype; SYNU-Ar00457.

#### Etymology.

The specific name refers to the type locality; noun in apposition.

#### Diagnosis.

The new species resembles *B.erromena* Zhang & Peng, 2011 (Zhang and Peng 2011: 58, fig. 7A–G) by having similar bulbal apophysis (distally hooked; Fig. 3C) and vulval pore plates (strongly curved, with several teeth; Figs 3B, 7B), but can be distinguished by procursus with prolatero-distal sclerite (arrow 1 in Figs 2C, 6A vs membranous lamella bearing comb-shaped apophyses, fig. 7D in Zhang and Peng 2011), by male cheliceral distal apophyses on distal part of chelicerae (da in Fig. 3D vs on submedian part, fig. 7B in Zhang and Peng 2011), and by epigynal pockets on postero-median part of epigynal plate and close to each other (ep in Figs 3A, B, 7A, B vs on anterior part of epigynal plate and widely separated, ep in fig. 7F in Zhang and Peng 2011).

#### Description.

**Male** (***holotype***): Total length 2.00 (2.10 with clypeus), carapace 0.72 long, 0.73 wide, opisthosoma 1.28 long, 0.89 wide. Leg I: 12.56 (3.28, 0.32, 3.13, 4.55, 1.28), leg II missing, leg III: 5.29 (1.51, 0.22, 1.25, 1.72, 0.59), leg IV: 7.19 (2.13, 0.25, 1.82, 2.30, 0.69); tibia I L/d: 41. Eye interdistances and diameters: PME–PME 0.14, PME 0.13, PME–ALE 0.02. Sternum width/length: 0.50/0.45. Habitus as in Fig. 3E, F. Carapace yellowish, without marks; clypeus and sternum yellowish. Legs whitish, without darker rings. Opisthosoma yellowish, without spots. Thoracic furrow absent. Clypeus frontally projected. Chelicerae with a pair of proximo-lateral apophyses (pa in Fig. 3D) and a pair of distal, prolaterally curved apophyses (distance between tips: 0.05; da in Fig. 3D). Palp as in Fig. 2A, B; trochanter with ventral apophysis (2× longer than wide; arrow 1 in Fig. 2B) and retrolateral apophysis (as long as wide; arrow 2 in Fig. 2B); femur with retrolateral protrusion (arrow 3 in Fig. 2B); procursus with fan-shaped prolatero-distal sclerite (arrow 1 in Figs 2C, 6A), distal membranous lamella (arrow 2 in Figs 2C, 6A), and retrolatero-subdistal membranous lamella (arrow in Figs 2D, 6B); bulb with hooked apophysis (ba in Fig. 3C) and distally bifurcated embolus (e in Fig. 3C). Retrolateral trichobothrium on tibia I at 5% proximally; legs with short vertical setae on metatarsi; tarsus I with 16 distinct pseudosegments.

**Female** (***paratype***, SYNU-Ar00457): Similar to male, habitus as in Fig. 3G, H. Total length 2.22 (2.32 with clypeus), carapace 0.70 long, 0.72 wide, opisthosoma 1.52 long, 1.10 wide. Leg I missing. Eye interdistances and diameters: PME–PME 0.12, PME 0.10, PME–ALE 0.02. Sternum width/length: 0.58/0.55. Clypeus unmodified. Epigynum oval, posteriorly curved, with postero-median pockets 0.03 apart (ep in Figs 3A, 7A). Vulva with anterior arch (aa in Figs 3B, 7B) and a pair of strongly curved pore plates with several teeth (pp in Figs 3B, 7B).

#### Variation.

Tibia I in two male paratypes (SYNU-Ar00454–55): 2.30, 3.00 (Leg I missing in SYNU-Ar00456).

#### Distribution.

China (Sichuan, type locality; Fig. 1).

### ﻿ Belisana tongjiang

Taxon classificationAnimaliaAraneaePholcidae

Wang, Li & Yao sp. nov.

C6B66EE4-1F51-5FA5-9D03-BBE6EF9ED4D5

https://zoobank.org/CFA56021-48BF-4FC0-9FD8-5F5D22D3BAF6

**Figure 4.**
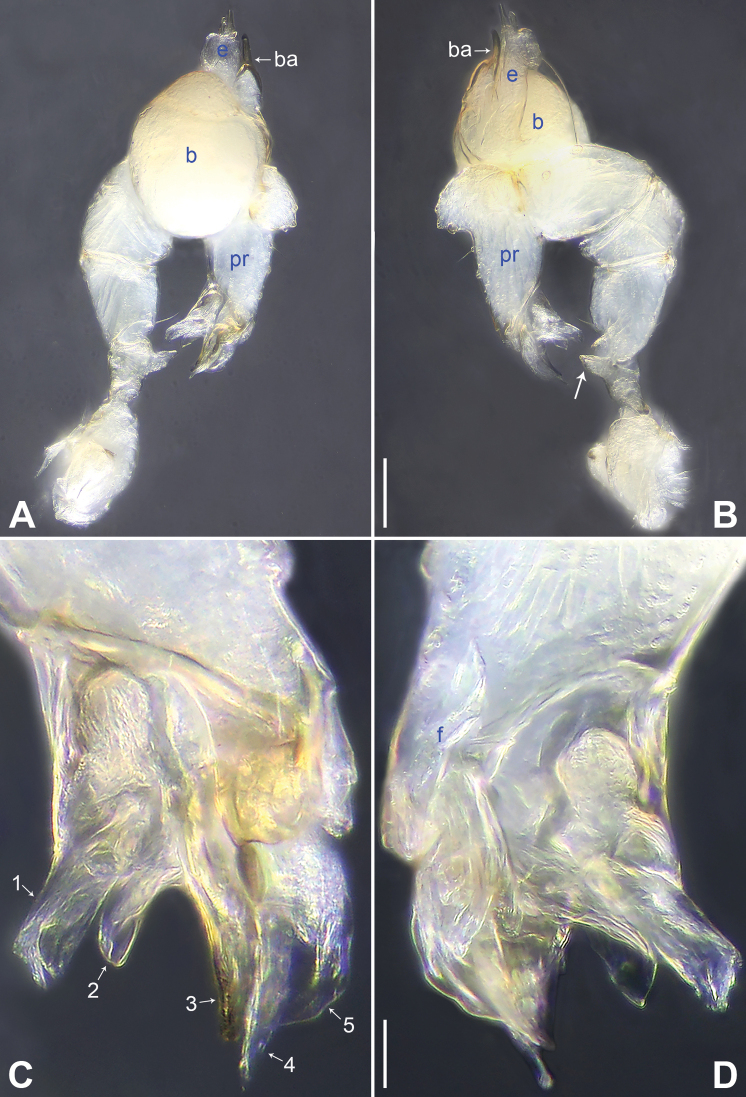
*Belisanatongjiang* sp. nov., holotype male **A, B** palp (**A** prolateral view **B** retrolateral view, arrow points at ventral apophysis) **C, D** distal part of procursus (**C** prolateral view, arrow 1 points at ventro-distal membranous process, arrow 2 points at distal membranous process, arrow 3 points at sclerotized distal apophysis, arrow 4 points at prolatero-distal membranous process, arrow 5 points at dorso-distal membranous process **D** retrolateral view). Abbreviations: b = bulb, ba = bulbal apophysis, e = embolus, f = flap, pr = procursus. Scale bars: 0.10 (**A, B**); 0.02 (**C, D**).

**Figure 5.**
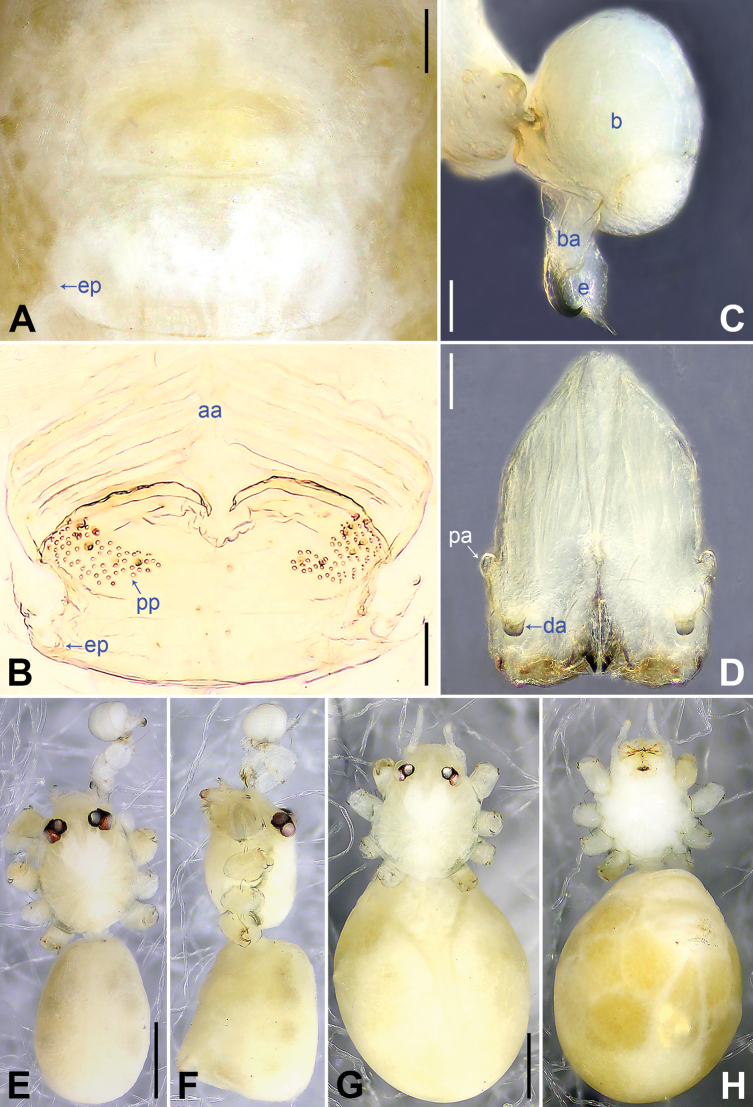
*Belisanatongjiang* sp. nov., holotype male (**C–F**) and paratype female (**A, B, G, H**) **A** epigynum, ventral view **B** vulva, dorsal view **C** bulb, prolateral view **D** chelicerae, frontal view **E–H** habitus (**E, G** dorsal view **F** lateral view **H** ventral view). Abbreviations: aa = anterior arch, b = bulb, ba = bulbal apophysis, da = distal apophysis, e = embolus, ep = epigynal pocket, pa = proximo-lateral apophysis, pp = pore plate. Scale bars: 0.05 (**A–D**); 0.30 (**E–H**).

**Figure 6.**
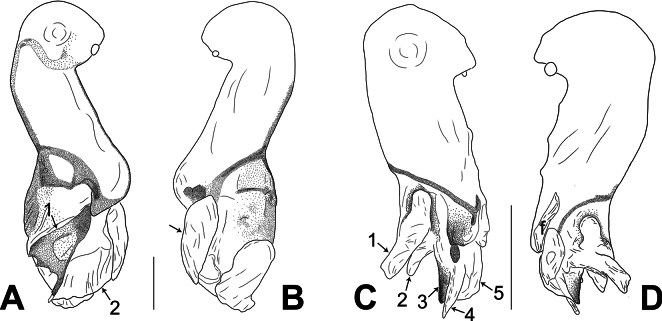
Procursus in prolateral and retrolateral views (arrows point at the same structures as those shown in the photos of each species) **A, B***Belisanamiyi* sp. nov. **C, D***B.tongjiang* sp. nov. Abbreviation: f = flap. Scale bars: 0.10.

**Figure 7.**
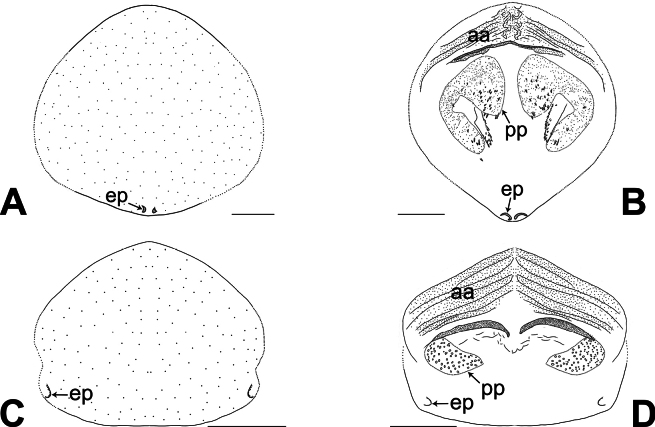
Female genitalia in ventral and dorsal views **A, B***Belisanamiyi* sp. nov. **C, D***B.tongjiang* sp. nov. Abbreviations: aa = anterior arch, ep = epigynal pocket, pp = pore plate. Scale bars: 0.10.

Figs 4 , 5 , 6C, D , 7C, D

#### Type material.

***Holotype*: China** • ♂; Sichuan, Bazhong, Tongjiang County, Nuojiang Town, Mulingzui Village; 31.892294°N, 107.217638°E; alt. 583 m; 21 Jun. 2024; X. Zhang, Y. Wang & Q. Meng leg.; SYNU-Ar00463. ***Paratypes*: China** • 5 ♀; same data as for holotype; SYNU-Ar00464–68.

#### Etymology.

The specific name refers to the type locality; noun in apposition.

#### Diagnosis.

The new species resembles *B.honghe* Zhang, Li & Yao, 2024 (Zhang et al. 2024b: 257, figs 2A–D, 3A–H, 18A, B, 20A, B) by having similar bulbal apophysis (distally hooked; Fig. 5C) and epigynum (epigynal pockets on postero-lateral part of epigynal plate; Figs 5A, B, 7C, D), but can be distinguished by procursus with distal membranous process (arrow 2 in Figs 4C, 6C vs sclerotized apophysis, arrow 2 in figs 2C, 18A in Zhang et al. 2024b), prolatero-distal membranous process (arrow 4 in Figs 4C, 6C vs absent, figs 2C, 18A in Zhang et al. 2024b) and retrolateral membranous flap (4× wider than long, f in Figs 4D, 6D vs 2× wider than long, f in figs 2D, 18B in Zhang et al. 2024b), by male cheliceral distal apophyses distally blunt (da in Fig. 5D vs distally pointed, da in fig. 3D in Zhang et al. 2024b), and by vulval pore plates nearly rectangular (pp in Figs 5B, 7D vs anteriorly pointed and posteriorly wide, pp in figs 3B, 20B in Zhang et al. 2024b).

#### Description.

**Male** (***holotype***): Total length 1.27 (1.38 with clypeus), carapace 0.45 long, 0.40 wide, opisthosoma 0.82 long, 0.50 wide. Legs I, II and IV missing, leg III: 4.42 (1.27, 0.18, 1.04, 1.44, 0.49). Eye interdistances and diameters: PME–PME 0.10, PME 0.07, PME–ALE 0.02. Sternum width/length: 0.43/0.40. Habitus as in Fig. 5E, F. Carapace yellowish, without marks; clypeus and sternum yellowish. Legs whitish, without darker rings. Opisthosoma yellowish, without spots. Thoracic furrow absent. Clypeus unmodified. Chelicerae with a pair of proximo-lateral apophyses (pa in Fig. 5D) and a pair of distal apophyses pointing downwards (distance between tips: 0.14; da in Fig. 5D). Palp as in Fig. 4A, B; trochanter with ventral apophysis (2× longer than wide; arrow in Fig. 4B); procursus with ventro-distal membranous process (arrow 1 in Figs 4C, 6C), distal membranous process (arrow 2 in Figs 4C, 6C), sclerotized distal apophysis (arrow 3 in Figs 4C, 6C), prolatero-distal membranous process (arrow 4 in Figs 4C, 6C), dorso-distal membranous process (arrow 5 in Figs 4C, 6C), and retrolateral membranous flap (f in Figs 4D, 6D); bulb with hooked apophysis (ba in Fig. 5C) and distally pointed embolus (e in Fig. 5C).

**Female** (***paratype***, SYNU-Ar00464): Similar to male, habitus as in Fig. 5G, H. Total length 1.48 (1.60 with clypeus), carapace 0.53 long, 0.56 wide, opisthosoma 0.95 long, 0.63 wide. Tibia I: 1.70; tibia I L/d: 22. Eye interdistances and diameters: PME–PME 0.12, PME 0.06, PME–ALE 0.02. Sternum width/length: 0.44/0.40. Epigynum nearly triangular, posteriorly straight, with postero-lateral pockets 0.25 apart (ep in Figs 5A, 7C). Vulva with anterior arch (aa in Figs 5B, 7D) and a pair of nearly rectangular pore plates (2× longer than wide; pp in Figs 5B, 7D).

#### Variation.

Tibia I in another female paratype (SYNU-Ar00465): 1.44 (Leg I missing in SYNU-Ar00466–68).

#### Distribution.

China (Sichuan, type locality; Fig. 1).

### ﻿ Belisana tongi

Taxon classificationAnimaliaAraneaePholcidae

Zhang, Li & Yao, 2024

E054B103-6D63-58C7-90CC-5EBE0BB9DB33

Fig. 8

Belisana tongi Zhang et al. 2024b: 273, figs 12A–D, 13A–H, 18K, L, 21C, D.

#### Material examined.

**China** • 2 ♂; Sichuan, Panzhihua, Miyi County, Puwei Town, Pengjiayakou Village; 27.060020°N, 102.000282°E; alt. 2464 m; 5 Jun. 2024; X. Zhang, Y. Wang & Q. Meng leg.; SYNU-Ar00458–59 • 3 ♀; same data as for preceding; SYNU-Ar00460–62.

**Figure 8.**
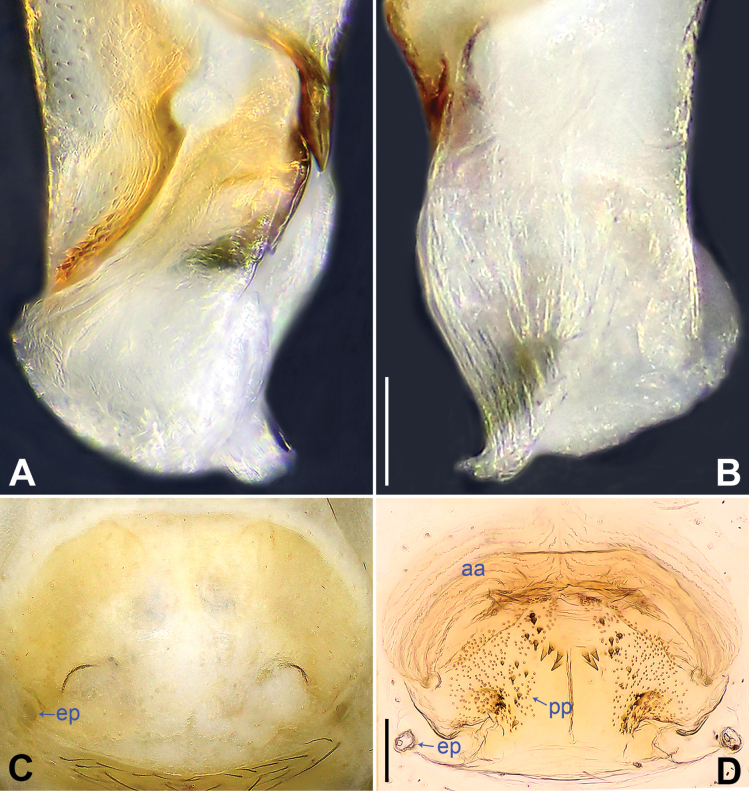
*Belisanatongi* Zhang, Li & Yao, 2024, male (**A, B**) and female (**C, D**) **A, B** distal part of procursus (**A** prolateral view **B** retrolateral view) **C** epigynum, ventral view **D** vulva, dorsal view. Abbreviations: aa = anterior arch, ep = epigynal pocket, pp = pore plate. Scale bars: 0.05 (**A, B**); 0.10 (**C, D**).

#### Distribution.

China (Sichuan, Fig. 1; Yunnan, type locality).

### ﻿ Belisana yanhe

Taxon classificationAnimaliaAraneaePholcidae

Chen, Zhang & Zhu, 2009

59522E08-DFAE-59B0-8B67-2EA53C8BA7ED

Fig. 9

Belisana yanhe Chen et al. 2009: 65, figs 31–38.

#### Material examined.

**China** • 1 ♂; Sichuan, Yaan, Lushan County, Feixianguan Town, Longdongpo; 30.090000°N, 102.930556°E; alt. 905 m; 24 May 2024; X. Zhang, Y. Wang & Q. Meng leg.; SYNU-Ar00469 • 1 ♀; same data as for preceding; SYNU-Ar00470.

**Figure 9.**
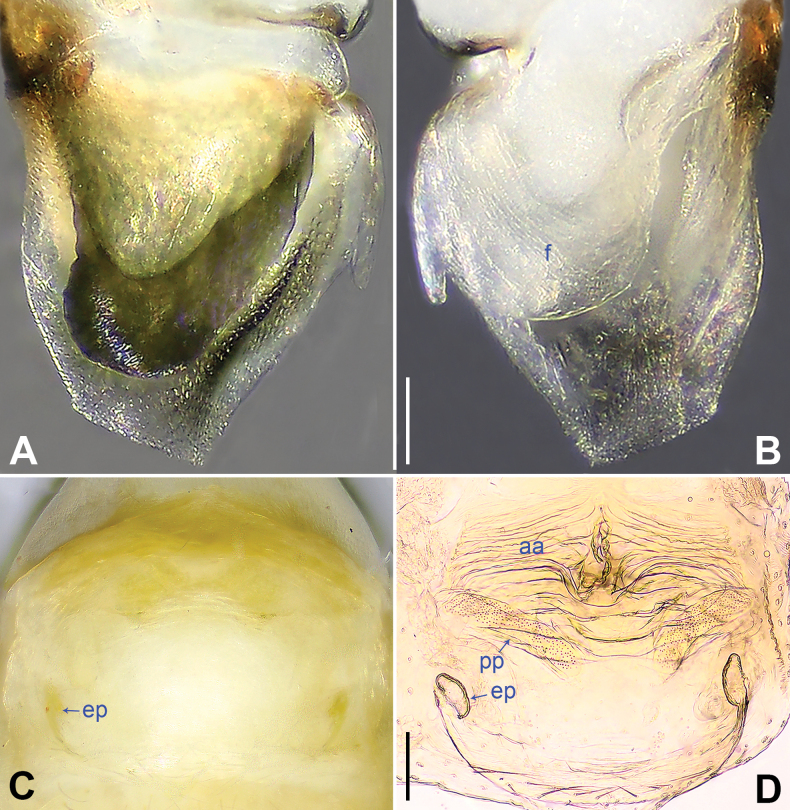
*Belisanayanhe* Chen, Zhang & Zhu, 2009, male (**A, B**) and female (**C, D**) **A, B** distal part of procursus (**A** prolateral view **B** retrolateral view) **C** epigynum, ventral view **D** vulva, dorsal view. Abbreviations: aa = anterior arch, ep = epigynal pocket, f = flap, pp = pore plate. Scale bars: 0.05 (**A, B**); 0.10 (**C, D**).

#### Distribution.

China (Sichuan, Fig. 1; Guizhou, type locality).

## Supplementary Material

XML Treatment for Belisana

XML Treatment for Belisana miyi

XML Treatment for Belisana tongjiang

XML Treatment for Belisana tongi

XML Treatment for Belisana yanhe

## ﻿Additional information

### Author ORCIDs

Bing Wang https://orcid.org/0009-0009-7047-4395

Ying Wang https://orcid.org/0009-0007-9173-4438

Shuqiang Li https://orcid.org/0000-0002-3290-5416

Pengfeng Wu https://orcid.org/0000-0001-5617-6708

Zhiyuan Yao https://orcid.org/0000-0002-1631-0949

### Data availability

All of the data that support the findings of this study are available in the main text.
